# Impact of incorporating electronic medical record tools on optimal antibiotic durations at discharge for uncomplicated community-acquired pneumonia: a quasi-experimental study

**DOI:** 10.1017/ash.2026.10326

**Published:** 2026-04-13

**Authors:** Merin Babu, Amy E. Beaulac, Janeen Dubay, Lori Leman, Anita B. Shallal, Rachel M. Kenney, Brian M. Church, Robert McCollom, Michael P. Veve, Sage B. Greenlee

**Affiliations:** 1 Department of Pharmacy, Henry Ford Macomb Hospital, USA; 2 Department of Pharmacy, Henry Ford West Bloomfield Hospital, USA; 3 Department of Quality, Henry Ford West Bloomfield Hospital, USA; 4 Department of Quality, Henry Ford Macomb Hospital, USA; 5 Department of Infectious Diseases, Henry Ford Hospital, USA; 6 Department of Pharmacy, Henry Ford Hospital, USA; 7 Epic Helios Pharmacy Team, Henry Ford Health, USA; 8 Department of Pharmacy Practice, Eugene Applebaum College of Pharmacy and Health Sciences, Wayne State Universtiy, USA

## Abstract

**Objective:**

To evaluate the impact of electronic medical record (EMR) transitions of care tools on antibiotic durations for uncomplicated community-acquired pneumonia (CAP).

**Design:**

IRB-approved, quasi-experiment.

**Setting:**

Five acute-care hospitals in Michigan.

**Patients:**

Hospitalized adults with uncomplicated CAP between 07/01/2023 and 11/30/2023 (pre-intervention) and 07/01/2024 and 11/30/2024 (post-intervention) were included. Patients were excluded if antibiotics were completed prior to discharge date, admitted to intensive care unit, respiratory culture with methicillin-resistant *Staphylococcus aureus* or *Pseudomonas aeruginosa* ≤12-months before admission, suspected concomitant infection, or complicated CAP.

**Methods:**

EMR tools implemented March–May 2024 included a total antibiotic days counter and an inpatient stop date carryover on discharge order. The primary outcome was the proportion of patients prescribed ≤6-calendar-days of therapy. Secondary outcomes included 30-day CAP-related readmission, *Clostridioides difficile* infection (CDI), multidrug-resistant organisms (MDRO) ≤90-days of discharge, and days of therapy prescribed at discharge.

**Results:**

234 patients were included: 124 pre- and 110 post-intervention. A higher proportion of post-intervention patients received ≤6-days of therapy (54% pre- vs 72.7% post-intervention, *P* = 0.003). No notable differences were seen in CDI or MDROs. Pre-intervention patients experienced more CAP-related readmissions (12.1% pre- vs. 4.5% post-intervention, *P* = 0.039) and more days of therapy at discharge [3-d (IQR 2–4) pre- vs. 2-d (IQR 1–4) post-intervention, *P* < 0.001]. After adjustment for confounders, the post-intervention group had 2-fold increased odds of receiving ≤ 6-days of therapy for CAP (adjOR, 2.27; 95%CI, 1.31–3.93).

**Conclusion:**

Implementation of EMR transitions of care tools significantly improved antibiotic durations in hospitalized adults with CAP, without negatively impacting patient outcomes.

## Background

Community-acquired pneumonia (CAP) is a common diagnosis amongst hospitalized patients; however, appropriate treatment remains a challenge. In a cross-sectional study of 192 US hospitals, Magill et al. found 79.5% (174/219) of patients diagnosed with CAP received unsupported treatment, with excessive antibiotic duration being a driving factor (103 of 174, [59.2%]).^1^ Historically, patients were treated with 10–14 days of antibiotics for CAP, but practice continues to evolve, as the 2025 American Thoracic Society (ATS) guidelines support a minimum duration of 3–5 days of antibiotic therapy for uncomplicated CAP compared to the 2019 ATS/Infectious Diseases Society of America guideline that recommends 5 days.^2–4^ Additionally, Dinh et al. note that discontinuing beta-lactam therapy after 3 days is non-inferior (clinical cure at day 15) to 8 days of treatment in clinically stable CAP patients.^5^ Thus, evidence for shorter durations continues to progress in uncomplicated CAP.

Antimicrobial stewardship programs strive to improve clinical outcomes by reducing inappropriate antimicrobial prescribing and antimicrobial resistance (AMR).^6^ Transitions of care continue to be difficult for antimicrobial stewardship efforts, with more than 1 in 8 inpatient admissions resulting in antimicrobial prescriptions at discharge.^6,7^ An estimated two-thirds of patients with CAP are treated with an excessive duration of antibiotics, where discharge prescribing represents a missed opportunity for intervention.^8^ Vaughn et al. found that extended treatment of CAP is associated with a higher proportion of patient-reported adverse events after discharge, without improvement in clinical outcomes.^8^ Overprescribing may also lead to AMR development and increased cost for the patient.^6^

To encourage appropriate antibiotic durations at discharge, the Henry Ford Health (HFH) antimicrobial stewardship program developed tools embedded within the electronic medical record (EMR). An antibiotic duration counter that displays within all inpatient discharge antibiotic prescriptions was created to assist prescribers with counting total calendar days of antibiotic therapy received during the inpatient hospitalization. This counter does not display if patients received zero days of antibiotics during their admission. Additionally, an automatic inpatient-to-discharge stop date carryover feature was enabled to facilitate inpatient order end dates continuing onto the outpatient antibiotic prescriptions generated during discharge medication reconciliation. Of note, placement of stop dates on inpatient antimicrobial orders is not required at HFH. This study sought to evaluate the impact of these interventions on antibiotic durations of therapy for CAP.

## Methods

### Study design and patient population

This Institutional Review Board (#17572-06) approved a prepost quasi-experimental study with a non-equivalent dependent variable was conducted at 5 acute care hospitals within HFH located in southeast Michigan. There were two study periods evaluated: July–November 2023 (preintervention) and July–November 2024 (postintervention). Adults (≥18 years old) admitted to 1 of the 5 hospitals during the preintervention or postintervention periods for uncomplicated CAP were eligible. CAP was considered uncomplicated if patients were clinically stable by discharge date or day 5 of therapy (whichever occurred first), were immunocompetent, and without structural lung disease, moderate or severe COPD, or documented pneumonia with methicillin-resistant *Staphylococcus aureus* (MRSA), methicillin-susceptible *Staphylococcus aureus* (MSSA), or *Pseudomonas aeruginosa* (PSA).^9^ Clinical stability was defined as patients without fever and without more than one of the following: heart rate >100 beats per minute, respiratory rate >24 breaths per minute, systolic blood pressure <90 mmHg, new onset altered mental status, or SpO2 < 90% or new oxygen requirement. Patients were excluded if they did not meet uncomplicated CAP criteria, if they completed antibiotics prior to the discharge date, were admitted to the intensive care unit during the encounter, had pulmonary complications (eg lung abscess, empyema, cavitations, parapneumonic effusions, loculations, necrotizing, or postobstructive pneumonia), were severely immunocompromised (AIDS/HIV, neutropenia (absolute neutrophil count <500 cells/µL), transplant history, ≥2 immunocompromising medications), or had suspected concomitant infections. Patients were identified and randomized for inclusion via the Michigan Hospital Medicine Safety (HMS) Consortium uncomplicated CAP patient list, which includes patients identified via ICD-10 codes for pneumonia. Data was extracted from the existing HMS database. Variables that could not be extracted from the HMS database were collected via retrospective chart review of the EMR using a standardized case report form.

### Intervention

Based on internal CAP prescribing data, in 2023, the HFH antimicrobial stewardship program developed an antibiotic duration workgroup who convened multiple meetings and presented suggested actions to the health system antimicrobial stewardship subcommittee. The workgroup solicited and identified 10 HFH prescribers to identify quality improvement ideas focused on duration of therapy workflows in CAP. Based on the results of this survey, provider workflow assessment, and feasibility evaluation with information technology pharmacy specialists, two CAP duration of therapy interventions were developed.

**Figure 1.**
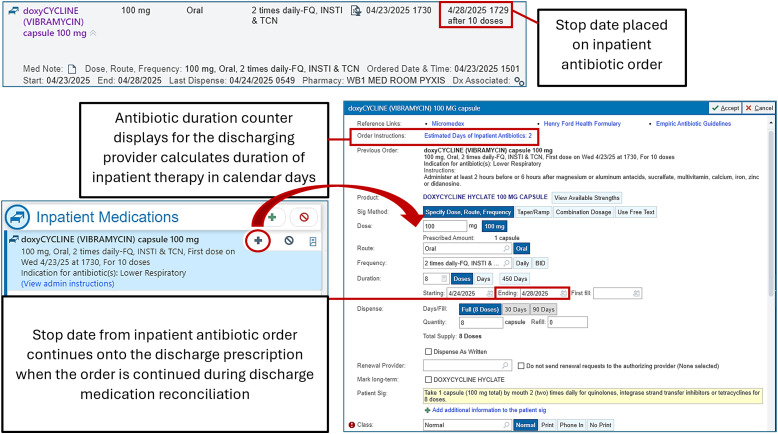
EMR transitions of care tools.

In March 2024, an antibiotic days of therapy counter was implemented within the EMR (Epic Systems Corporation, Madison, WI, USA). The count appears within all inpatient discharge antibiotic prescriptions and displays the total calendar days of antibiotic therapy administered during the inpatient encounter (Figure 1). In May 2024, a stop date carryover was established to facilitate end-of-therapy continuity from inpatient to outpatient antibiotic prescribing (Figure 1). With this functionality, a stop date placed on an inpatient medication order automatically carries over to the outpatient prescription of the same medication when the order is continued at discharge medication reconciliation.

### Outcomes

The primary outcome was to compare the proportion of patients who were prescribed ≤6 days of antibiotic therapy for CAP. Six days of therapy was chosen as the evaluation point to account for prescriber consideration of antibiotic doses compared to that of calendar days. Secondary outcomes were to compare 30-day CAP-related readmissions, incidence of *Clostridioides difficile* infections 30 days after discharge, number of patients with multidrug-resistant organism (MDRO) isolates within 90 days after discharge, and number of antibiotic days of therapy prescribed at discharge for CAP amongst the comparator groups.

### Statistical analysis

Based on 2023 HMS data, an estimated 66% of patients admitted to HFH with uncomplicated CAP received ≤6 days of total antibiotic therapy. Therefore, a total of 270 patients, with 135 in each period, were estimated to achieve an alpha of 0.05 with 80% power to detect a 15% difference between groups. Descriptive statistics were used to summarize patient demographics in the pre and postintervention groups. χ^2^ or Fisher’s exact tests were applied to categorical variables and results expressed as percentages. Continuous variables were analyzed using the Mann-Whitney U test, assuming a non-parametric distribution, and results were reported as medians with interquartile ranges. Test significance was considered if the *P*-value was <.05. To determine variables independently associated with prescribing ≤6 days of antibiotic therapy for CAP, variables that were found to be associated with the primary outcome from bivariate analysis were entered into a multivariable logistic regression model using a backward, stepwise approach. The model incorporated variables with a *P* < .2 and/or displaying clinical relevance. Categorical variables were assessed for collinearity using the χ^2^ test. Model fit was assessed using the Hosmer-Lemeshow goodness-of-fit test. A non-equivalent dependent variable of fluoroquinolone prescribing at any point in the CAP treatment course was used to evaluate the standard of care over the study period and evaluate for maturation effect, as the decision to prescribe or not prescribe a fluoroquinolone at any point in the course of treatment (vs other antibacterial agents) would not be impacted by the study intervention. Statistical analysis was performed with SPSS Statistics, version 29 (IBM Corp., Armonk, NY, USA).

## Results

**Figure 2.**
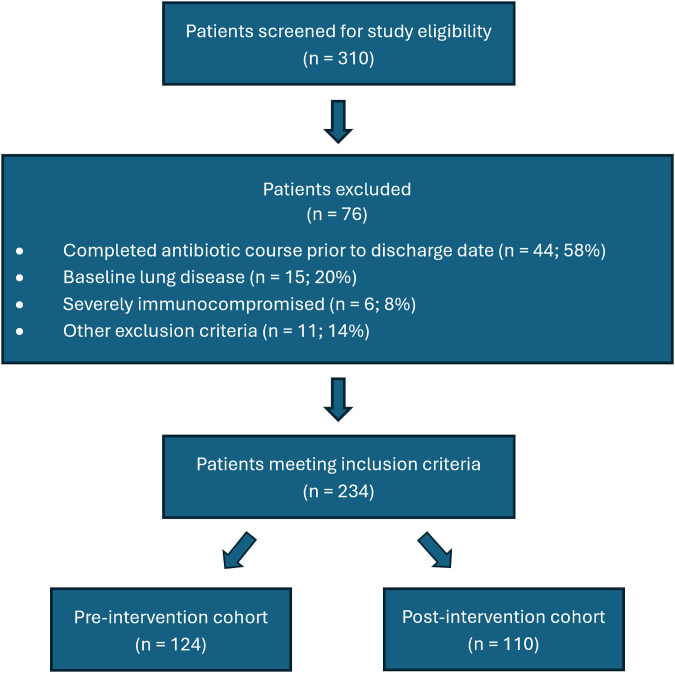
Inclusion and exclusion of patients.

A total of 310 patients were screened, with 234 patients meeting inclusion criteria—124 patients in the pre-intervention period and 110 patients in the post-intervention period. Of those excluded, 44 (58%) completed antibiotics prior to their discharge date, 15 (20%) had baseline lung disease, 6 (8%) were severely immunocompromised, and 11 (14%) met other exclusion criteria (Figure 2). Baseline demographics are summarized in Table 1. Treatment groups were well-balanced, although there were significantly more white/Caucasian patients in the post-intervention group (66.9% pre- vs 79.1% post-intervention, *P* = .037). The median (IQR) inpatient antibiotic duration was significantly shorter in the pre-intervention group [3 days (3–4) vs 4 days (3–5), *P* = .027], and while median (IQR) length of stay was 3 days in both groups, there was a difference in distribution between the pre- and post-intervention periods [3 days (2–3) vs 3 (2–4), *P* = .003]. More patients with a beta-lactam allergy were in the pre-intervention group compared to the post-intervention group (28.2% vs 21.8%, *P* = .260). Atypical agents, including fluoroquinolones, tetracyclines, or macrolides, were the most prescribed antibiotic coverage in both inpatient and outpatient settings amongst either group (Table 2). In addition, there were significantly more patients discharged on penicillin agents (eg amoxicillin, amoxicillin/clavulanate) in the pre-intervention period (55.6% pre- vs 40% post-intervention, *P* = .017). The non-equivalent dependent variable suggested a modest magnitude of maturation and reduction in fluoroquinolone prescribing, 15.3% pre- versus 6.4% post-intervention. Based on further analysis, 13 of 19 patients prescribed a fluoroquinolone had a beta-lactam allergy in the pre-intervention period, compared to 1 of 7 patients in the post-intervention period (68.4% pre- vs 14.3% post-intervention, *P* = .030). Alternatively, 37.1% of beta-lactam allergic patients received a fluoroquinolone in the pre-intervention compared to only 4.2% in the post-intervention.

**Table 1.** Demographics of patients with community acquired pneumonia before and after implementation of EMR duration of therapy interventions

Variable, *n* (%) or median (IQR)	Pre-intervention (*n* = 124)	Post-intervention (*n* = 110)	*p*-value
Age, years	66.5 (54.3–79)	71 (54.8–82)	.229
Sex, male	74 (59.7)	52 (47.3)	.057
Race, white/Caucasian	83 (66.9)	87 (79.1)	.037
Language, English	116 (93.5)	107 (97.3)	.225
BMI, kg/m^2^	28.2 (24.3–34.5)	28.2 (24.6–34)	.895
Insurance, public	73 (58.9)	58 (52.7)	.345
CURB-65			
0-1	58 (46.8)	43 (39.1)	.236
2	41 (33.1)	38 (34.5)	.811
≥3	25 (20.2)	29 (26.4)	.261
COPD, mild	24 (19.4)	22 (20)	.901
Beta-lactam allergy	35 (28.2)	24 (21.8)	.260
Antibiotics within 90 days	15 (12.1)	13 (11.8)	.948
Hospitalization within 90 days	20 (16.1)	14 (12.7)	.461
ID consultation	15 (12.1)	13 (11.8)	.948
Inpatient antibiotic duration, days	3 (3–4)	4 (3–5)	.027
Length of stay, days	3 (2–3)	3 (2–4)	.003

Note. BMI, body mass index; COPD, chronic obstructive pulmonary disease; ID, infectious disease.

**Table 2.** Antibiotic classes and spectrum used in the treatment of uncomplicated community acquired pneumonia

Variable, *n* (%)	Pre-intervention (*n* = 124)	Post-intervention (*n* = 110)	*p*-value
Inpatient therapy			
Anti-MRSA	14 (11.3)	9 (8.2)	0.425
Antipseudomonal	12 (9.7)	8 (7.3)	0.511
Atypical	119 (96)	110 (100)	0.062
Cephalosporin*	47 (37.9)	46 (41.8)	0.541
Penicillin*	80 (64.5)	62 (56.4)	0.203
Outpatient Therapy			
Atypical	101 (81.5)	85 (77.3)	0.429
Cephalosporin*	24 (19.4)	24 (21.8)	0.641
Penicillin*	69 (55.6)	44 (40)	**0.017**
Received fluoroquinolone at any point	19 (15.3)	7 (6.4)	**0.030**

* No coverage for *Pseudomonas aeruginosa*.

Regarding the primary outcome, patients in the post-intervention period were more likely to be prescribed *≤*6 days of total antibiotic therapy (54% pre vs 72.7% postintervention, *P* = .003). Secondary outcomes are presented in Table 3. No notable differences were seen in *C. difficile* infections or MDRO development between the groups. In contrast, there were significantly more patients in the pre-intervention period who experienced CAP-related readmission within 30 days of discharge (12.1% pre- vs 4.5% post-intervention, *P* = .039). There were also significantly less days of therapy prescribed at discharge in the post-intervention group [3 (IQR 2–4) pre- vs 2 (IQR 1–4) post-intervention, *P* < .001].

**Table 3.** Secondary outcomes of patients with community acquired pneumonia before and after implementation of EMR duration of therapy interventions

Variable, *n* (%) or median (IQR)	Pre-Intervention (*n* = 124)	Post-Intervention (*n* = 110)	*p*-value
30- day CAP-related readmission	15 (12.1)	5 (4.5)	**.039**
Incidence of *C. difficile* 30 days after discharge	1 (0.8)	0 (0)	1.000
Patients with MDRO isolates within 90 days after discharge	1 (0.8)	2 (1.8)	.602
Days of antibiotic therapy prescribed at discharge	3 (2–4)	2 (1–4)	**<.001**

Note. CAP, community acquired pneumonia; MDRO, multidrug-resistant organism.

The results of the bivariate analyses and clinical rationale dictated the variables selected for inclusion into a multivariable logistic regression model (Table 4). This included patients who received outpatient treatment with non-fluoroquinolone therapy and patients in the post-intervention group exposed to the transitions of care tools intervention. The variables infectious disease (ID) consultation and CURB-65 score >2 were excluded from the model due to unmet statistical criteria. In the final model, patients in the post-intervention group had 2-fold increased odds of receiving ≤6 days of antibiotic therapy for CAP (adjOR, 2.47; 95%CI, 1.31–3.93).

**Table 4.** Variables associated with prescribing ≤6 days of antibiotic therapy for CAP

Variable	Unadjusted OR (95% CI)	*p*-value	Adjusted OR (95% CI)	*p*-value
PostIntervention group	2.27 (1.31–3.93)	.001	2.27 (1.31–3.93)	**.003**
Outpatient treatment with non-fluoroquinolone	1.98 (0.833–4.70)	.117	1.75 (0.72–4.23)	.21
ID Consultation	0.68 (0.31–1.50)	.34	Not tested	Not tested
CURB-65 Score >2	0.892 (0.52–1.52)	.673	Not tested	Not tested

Note. CAP, community acquired pneumonia; CURB-65, confusion, urea, respiratory rate, blood pressure, age ≥ 65; ID, Infectious Disease.

Hosmer-Lemeshow goodness-of-fit test results; Chi-square, 0.052; *P* = .974.

## Discussion

After implementation of EMR transitions of care prescribing tools, adult patients were 2-fold more likely to receive ≤6 days of antibiotic therapy for uncomplicated CAP. This finding displays the effectiveness of EMR-based interventions in aligning antibiotic duration with guideline recommendations. Additionally, patients exposed to the interventions received significantly less days of therapy at discharge; however, it should be noted that these patients also had significantly more inpatient antibiotic days which is unrelated to the intervention purpose.

Gaps identified in antibiotic prescribing during transitions of care highlight the need for antimicrobial stewardship interventions aimed at optimizing duration of therapy. As a participant of the Michigan HMS Consortium, our health system has the opportunity to collaborate with other health systems across the state to improve care for hospitalized patients. One HMS collaborative quality initiative is optimizing antimicrobial use in uncomplicated CAP by reducing the duration of antibiotic treatment for uncomplicated CAP to 5 days.^9^ Based on prior research assessing antibiotic treatment durations for pneumonia in Michigan, 93.2% of patients were prescribed an excess duration at discharge.^8^ Another study conducted in 2018 assessing average length of therapy of antibiotics for uncomplicated CAP showed that antibiotic length of therapy exceeded recommended duration of therapy in 72% of patients.^10^ These findings support the need for antimicrobial stewardship in all phases of care. As inpatient antimicrobial stewards continue to face high bed-to-staff ratios, programs have leaned into leveraging the EMR to assist in antimicrobial stewardship efforts, such as microbiology comment nudges, automatic antibiotic stop dates, diagnostic stewardship interventions, amongst others.^11–13^ Additionally, recent articles have highlighted the role of pharmacists in optimizing antibiotic use at discharge; however, many institutions are unable to allocate pharmacist time for discharge medication review due to excessive pharmacist-to-patient ratios.^14–16^ This study adds a unique approach to optimizing antimicrobial use upon discharge and further contributes to the growing practice of leveraging the EMR as an essential tool in antimicrobial stewardship efforts.

This study has several limitations. First, it is subject to maturation effect. While possible, there were no targeted campaigns toward CAP durations of therapy outside of day-to-day established efforts by the stewardship team. Educational efforts were conducted around use of the transitions of care tools for total antibiotic durations of therapy, for which a washout period was included to account for this time. Of note, the non-equivalent dependent variable of fluoroquinolone use was different between the groups (15.3% pre vs 6.4% post-intervention, *P* = .030). Upon further analysis, more patients with beta-lactam allergies received fluoroquinolones in the pre-period compared to the post-period. In the fall of 2024, there was a system-wide antimicrobial stewardship initiative to provide education on beta-lactam allergies and safe cephalosporin use. These efforts likely contributed to less fluoroquinolone prescribing in the post-intervention group, explaining the difference in the non-equivalent dependent variable. We do not anticipate these variations in fluoroquinolone prescribing impacted the study outcomes. Additionally, there is no method to ensure these tools were being referenced for all patients at discharge even with providers being educated on the tools and utility, noting that stop dates are not required on antibiotics at HFH. Other limitations of this study include the period for inclusion did not include several months of typical “respiratory season,” which could have limited cases available for inclusion; appropriateness of antibiotic selection was not assessed given most CAP therapy is empiric and the absence of microbiologic testing to drive therapy; and uncomplicated CAP diagnosis was not based on traditional definition of less than 3 or minor criteria, as noted in the 2025 ATS guidelines, but instead based on clinical stability, CURB-65 score, and exclusion of complicated patients as noted above.^3^ Additionally, despite efforts to obtain adequate sample size, the study population was limited and is at risk of Type I error given the conclusion of significance of the primary end point, as the estimated sample size was not met to adequately power the study. Further studies evaluating the impact of EMR transitions of care tools on antibiotic durations of therapy would be valuable.

In summary, adult patients were 2-fold more likely to receive appropriate durations of therapy for uncomplicated CAP after the implementation of EMR transitions of care tools, without negatively impacting patient outcomes. Future studies should continue to explore EMR functionality to optimize antibiotic prescribing practices at transitions of care.
